# Subarachnoid Hemorrhage From Ruptured Pseudoaneurysm Secondary to Dissection of the Anterior Cerebral Artery: A Case Report

**DOI:** 10.7759/cureus.85275

**Published:** 2025-06-03

**Authors:** David Norman L Fuentes, Charlene Mary C Mercado, Jay Villavicencio, Manuel M Mariano

**Affiliations:** 1 Neurosurgery, St. Luke's Medical Center, Quezon City, PHL

**Keywords:** anterior communicating artery, arterial dissection, embolization, pseudoaneurysm, subarachnoid hemorrhage

## Abstract

Arterial dissections occur when blood enters and separates the layers of the arterial wall. They are typically classified as either subintimal dissections, which are associated with ischemia or infarction, or subadventitial dissections, which can lead to subarachnoid hemorrhage (SAH).

Dissections most commonly occur extradurally, particularly in the extracranial segments of the carotid and vertebral arteries. When intradural, they tend to involve the distal vertebral arteries and the supraclinoid internal carotid artery (ICA). Less commonly, dissections affect the anterior cerebral artery (ACA) and middle cerebral artery (MCA).

Treatment options include endovascular approaches such as coil embolization and flow diversion, as well as open cerebrovascular techniques, including parent vessel sacrifice and vessel trapping. However, due to the rarity of ACA dissections, particularly in the A1 segment, there are no established treatment guidelines.

We present a rare case of an ACA-A1 segment dissection successfully treated with stent-assisted coil embolization, resulting in complete recovery.

## Introduction

Arterial dissection results from a tear in the intimal layer of the vessel wall, allowing blood to enter the arterial wall and create a false lumen. This can lead to luminal stenosis, occlusion, or pseudoaneurysm formation [1]. While spontaneous arterial dissections are more commonly seen in the extracranial segments of the internal carotid and vertebral arteries, advances in neuroimaging have led to increased recognition of spontaneous intracranial arterial dissection (SICAD). Intracranial dissections more frequently involve the posterior circulation, particularly the vertebrobasilar arteries, whereas involvement of the anterior cerebral artery (ACA), middle cerebral artery (MCA), and posterior cerebral artery (PCA) is relatively rare [2].

Dissections may occur spontaneously or following trauma and can present clinically with ischemia, subarachnoid hemorrhage (SAH), or mass effect on adjacent structures. Headache and posterior neck pain are common initial symptoms in spontaneous dissections [2]. In a study by Nagamine et al. (2014), patients with isolated ACA territory infarction secondary to dissection were typically younger, more likely to be physically active at stroke onset, and presented more often with headache. These patients also had higher initial blood pressure (BP) and lower D-dimer levels, and generally experienced more favorable clinical outcomes [3].

Diagnosis of ACA dissection relies on a combination of clinical features and neuroimaging findings. Key diagnostic indicators include the sudden onset of ischemic or hemorrhagic symptoms, cerebral angiographic findings corresponding to the clinical presentation, and hallmark imaging signs of dissection such as a double lumen or intimal flap, focal stenosis with post-stenotic dilation (the “pearl and string” sign), tapered narrowing (the “string” sign), or complete occlusion [4].

Management strategies are guided by the severity of symptoms and radiographic findings. Endovascular interventions such as stenting, coiling, or flow diversion are increasingly employed, particularly in cases involving hemorrhagic complications. Otaki et al. (2021) described a case in which a 67-year-old woman with severe SAH due to ACA dissection was successfully treated with multiple neck-bridge stents in the acute phase, facilitated by prasugrel, a third-generation antiplatelet agent with low resistance [5]. In selected cases, especially those with growing aneurysms or poorly controlled hypertension, open surgical options such as parent artery trapping, vessel sacrifice, bypass grafting, or resection of the dissected segment may be considered. For instance, Wakabayashi et al. (2000) reported favorable neurological outcomes following surgical trapping of a dissecting ACA aneurysm [6].

The natural history of arterial dissection is significantly influenced by its initial clinical presentation. When dissection presents with ischemic symptoms such as stroke or transient ischemic attack, the prognosis is generally favorable. Spontaneous cervical artery dissections causing ischemia have a reported 75% rate of good recovery, with less than 5% mortality. The highest risk of stroke occurs in the first few weeks following dissection onset but declines thereafter. Over time, many dissected arteries undergo spontaneous healing and recanalization, and dissecting aneurysms in these cases rarely rupture [7]. In contrast, dissections that present with SAH tend to follow a more aggressive clinical course. Intracranial dissections causing SAH are associated with high morbidity and mortality due to rapid bleeding into the subarachnoid space. Pathologically, these cases often involve full-thickness arterial wall disruption without compensatory remodeling, increasing the risk of rupture and complicating clinical management [8].

## Case presentation

Our patient is a 26-year-old Filipino man with no known comorbidities who presented to the emergency department following an acute neurological event. Approximately one hour prior to consultation, he experienced a sudden-onset, severe headache, which was immediately followed by a generalized tonic-clonic seizure lasting approximately two minutes. Postictally, the patient was noted to be unresponsive, prompting urgent transport to our facility for evaluation.

Upon arrival at the emergency department, the patient had a Glasgow Coma Scale (GCS) score of 3 (E1V1M1). He exhibited no purposeful movement and had irregular respirations. Pupils were 3 mm bilaterally and sluggishly reactive to light. Vital signs were notable for hypertension (BP: 170/100 mmHg) and bradycardia (heart rate (HR): 50 bpm), raising concern for increased intracranial pressure. Given his poor neurological status and lack of airway protective reflexes, the patient was immediately intubated for airway protection and ventilatory support.

**Figure 1:**
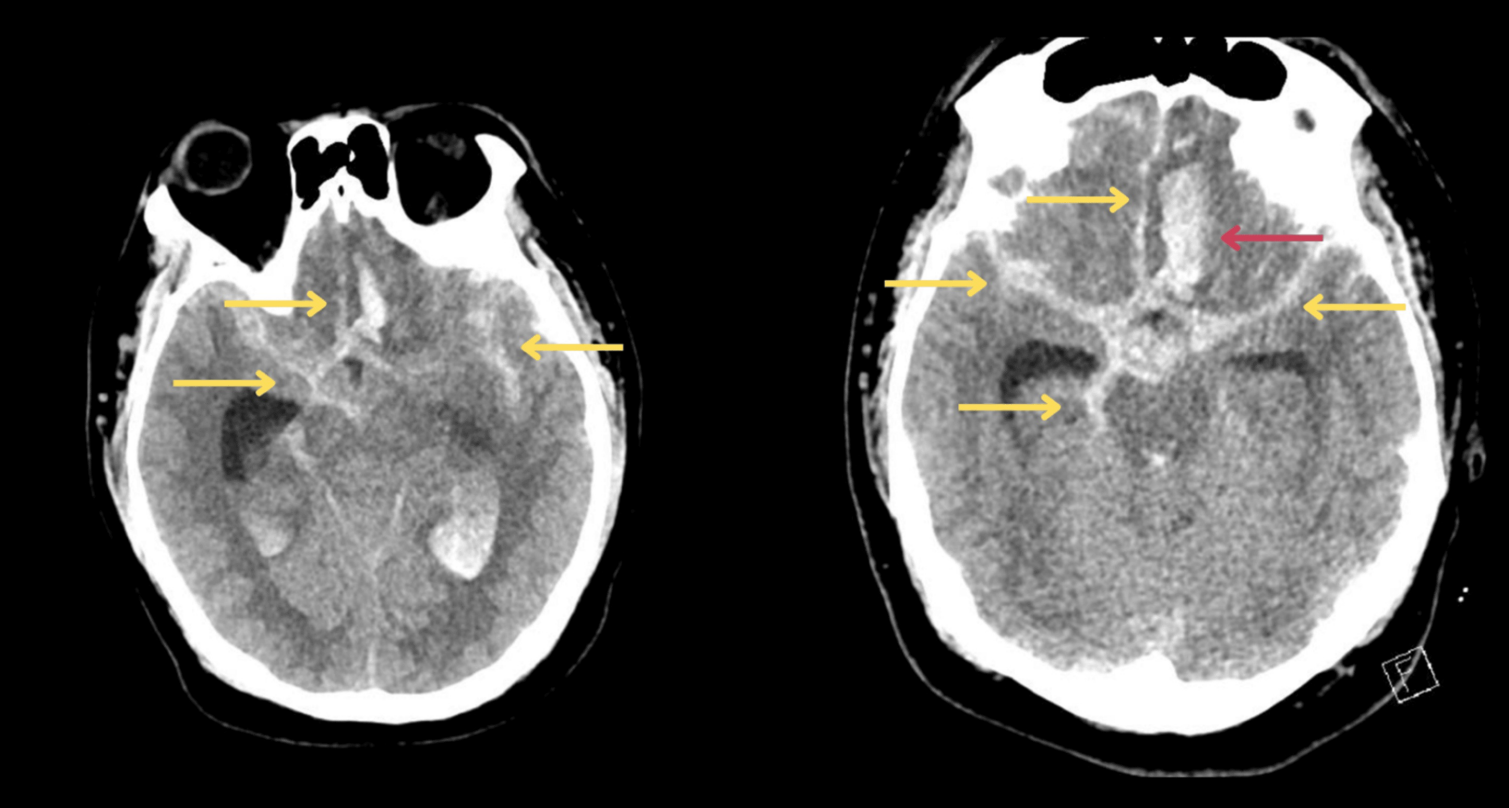
Plain cranial CT scan done one hour post-ictus showing diffuse subarachnoid hemorrhage (yellow arrows) with intracerebral hemorrhage (red arrow) CT: computed tomography

A non-contrast cranial computed tomography (CT) scan performed approximately one hour post-ictus demonstrated diffuse subarachnoid hemorrhage (SAH), predominantly involving the perimesencephalic cisterns, interhemispheric fissure, and bilateral Sylvian fissures and extending into the bilateral frontoparietal cortical sulci, consistent with high-grade SAH (Fisher grade 4). In addition, there was evidence of a left frontal intracerebral hemorrhage (ICH) measuring approximately 12 mL in volume, without midline shift or evidence of herniation at the time of imaging. No hydrocephalus was observed on initial imaging (Figure 1).

**Figure 2:**
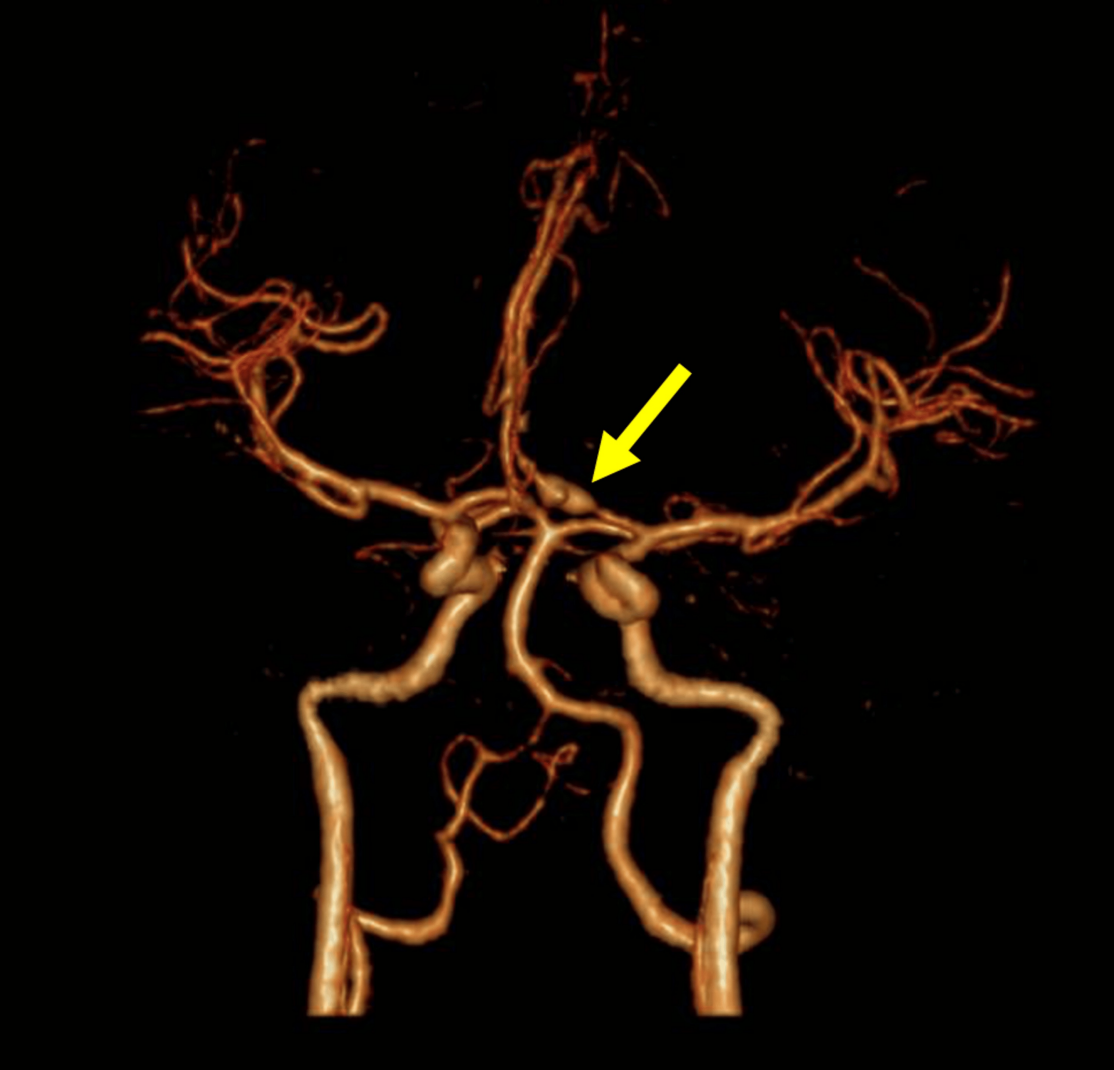
Cranial CT angiogram CT: computed tomography

This constellation of findings was concerning for a ruptured intracranial aneurysm or arterial dissection with associated pseudoaneurysm formation, prompting further vascular imaging for definitive diagnosis and treatment planning. The cranial CT angiogram showed an area of stenosis followed by a focal dilatation in the left ACA-A1 branch, which was suspicious for an arterial dissection (Figure 2).

**Figure 3:**
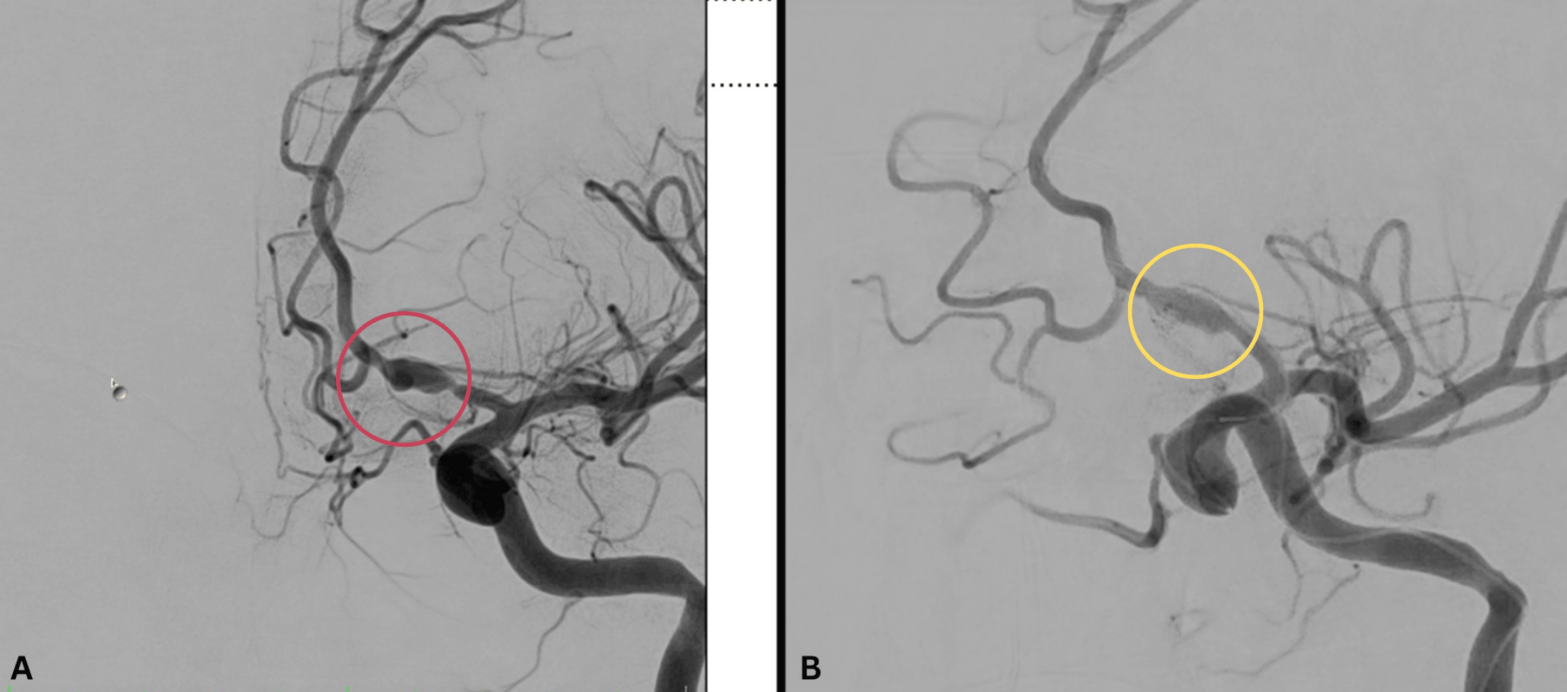
(A) Control angiogram pre-coiling showing the dissection on the left ACA-A1 segment, with an associated pseudoaneurysm (encircled in red) versus (B) post-coiling (encircled in yellow) ACA: anterior cerebral artery

A diagnostic cerebral catheter angiography was performed with preparation for potential endovascular intervention. Angiographic injections confirmed an arterial dissection involving the left anterior cerebral artery (ACA) A1 segment, with an associated pseudoaneurysm. We proceeded with stent-assisted coil embolization of the pseudoaneurysm. Prior to the procedure, the patient received 240 mg of aspirin and 300 mg of clopidogrel for antiplatelet loading. Three platinum coils were deployed, and follow-up angiography demonstrated complete obliteration of the pseudoaneurysm with preservation of flow in the parent artery (Figure 3).

The patient’s neurological status showed gradual and consistent improvement throughout the course of his hospitalization. By the end of the first week, he was weaned off mechanical ventilation and extubated successfully, with progressive recovery in consciousness and motor responses. Repeat cranial imaging showed stable findings, with no evidence of rebleeding or hydrocephalus. He was closely monitored in the neurocritical care unit, followed by step-down care in a neurology ward.

During his 23-day hospital stay, he underwent multidisciplinary rehabilitation, including physical and occupational therapy. He demonstrated marked improvement in cognitive function, coordination, and strength, regaining the ability to ambulate independently and perform self-care tasks.

At the outpatient follow-up conducted one month post-discharge, the patient had returned to his baseline neurological status. He was fully oriented, cognitively intact, and independent in all activities of daily living (ADLs), including personal hygiene, feeding, ambulation, and communication. He reported no residual headaches, focal deficits, or seizure recurrence, and had resumed normal functioning.

## Discussion

Arterial dissections occur as blood extends between the layers of the arterial wall. They can be traumatic (seen in 40%) or spontaneous [9]. Associated conditions and risk factors include the following: (1) connective tissue disorders, (2) vascular conditions such as Ehlers-Danlos syndrome and fibromuscular dysplasia, and (3) various conditions such as hypertension, migraine, oral contraceptive use, and smoking [10]. In North America and Europe, extracranial dissection is far more frequent, whereas in the Asian population, intracranial dissection is more common. About 88% of arterial dissections are extradural, commonly in the extracranial carotid arteries, 2-3 cm distal to the carotid bulb. Another common extradural location is the V2 or the V3 segment of the vertebral artery. Roughly 12% of arterial dissections are intradural, more common in the posterior circulation than the anterior circulation. For intradural locations, they are more commonly seen in the supraclinoid ICA, whereas less common locations include the anterior cerebral artery and middle cerebral artery [11].

The arterial wall is composed of three layers: the tunica intima, which is the lining of the endothelium; the tunica media, corresponding to a middle muscular layer; and the tunica adventitia, which corresponds to longitudinally arranged collagen fibers. Arterial dissection can arise from a tear in the tunica intima. The tear allows blood to enter the wall of the artery, creating a false lumen. The activation of platelets at the site of the intimal tear may lead to thrombus formation, which can be a cause of thromboembolic phenomena. Arterial dissection may either be a (1) subintimal dissection, in which there is stenosis of the anatomic arterial lumen and is commonly associated with symptoms of ischemia/infarct, or a (2) subadventitial dissection, which causes an aneurysmal dilation of the arterial wall and is prone to cause subarachnoid hemorrhage. Subadventitial dissection is commonly associated with pseudoaneurysms, which are dilatations in the vessel wall (composed of only media and adventitia) that are prone to hemorrhage [9-11].

The prognosis of intracranial arterial dissections is closely linked to the location of the mural tear and the type of dissection, whether subintimal or subadventitial. Subintimal dissections often present with luminal narrowing and ischemic symptoms, and typically have a favorable prognosis when patients are promptly started on antiplatelet therapy. In contrast, subadventitial dissections are more likely to rupture into the subarachnoid space, presenting as subarachnoid hemorrhage (SAH), and are associated with a significantly worse prognosis, with reported mortality rates reaching up to 50% [12,13]. Among patients presenting with SAH due to arterial dissection, the risk of rebleeding can be as high as 50% within the first 14 days, emphasizing the importance of early diagnosis and timely intervention [14].

In cases of suspected SAH, as with the current case, initial diagnostic imaging should include non-contrast computed tomography (CT) followed by CT angiography (CTA) to evaluate for vascular abnormalities. Additional imaging modalities such as magnetic resonance imaging (MRI), particularly T1-weighted fat-suppressed sequences, and magnetic resonance angiography (MRA) can aid in identifying features of dissection. Dissections may present on MRA as irregular luminal narrowing with or without post-stenotic dilatation (referred to as the “string sign” or “pearl and string sign”), as well as the presence of a double lumen or intimal flap [15]. Digital subtraction angiography (DSA) remains the gold standard for definitive diagnosis, providing detailed visualization of key angiographic features such as the string sign, dissecting pseudoaneurysm, and intimal flap.

Management strategies depend on the clinical presentation. In arterial dissections presenting with ischemic symptoms, conservative treatment with antiplatelet therapy is typically favored, and patients are monitored for spontaneous healing and recanalization. However, conservative management is contraindicated in patients presenting with SAH due to arterial dissection, given the high risk of rebleeding and poor clinical outcomes. For ruptured dissections with associated pseudoaneurysms, both open surgical and endovascular treatments are considered. Open surgical options include parent vessel sacrifice, trapping with or without bypass, and vessel wrapping. Endovascular approaches encompass coil embolization, stent-assisted coiling, and the use of flow-diverting stents, which have shown promise in reconstructing the parent artery while excluding the pseudoaneurysm [16].

In the present case, endovascular stent-assisted coiling was performed to occlude the pseudoaneurysm while preserving flow through the anterior cerebral artery (ACA). Given the rarity of ACA dissections, there is insufficient evidence to establish the superiority of either open or endovascular management. Thus, treatment decisions should be individualized, taking into account the patient’s clinical status, aneurysm morphology, and institutional expertise.

## Conclusions

Intracranial arterial dissections represent a critical and potentially life-threatening vascular pathology, especially when presenting as subarachnoid hemorrhage. The clinical behavior depends largely on the location and type of dissection. Subintimal dissections generally result in ischemic events and carry a more favorable prognosis, whereas subadventitial dissections are prone to rupture and are associated with high morbidity and mortality. Accurate and timely diagnosis, using a combination of non-invasive and catheter-based imaging modalities, is vital in guiding appropriate management. While conservative treatment with antiplatelet therapy may be sufficient for ischemic presentations, hemorrhagic cases often necessitate urgent intervention. In the absence of established guidelines for rare dissection sites such as the anterior cerebral artery, treatment strategies, whether surgical or endovascular, must be tailored to individual patient factors and available expertise. Our case highlights the role of stent-assisted coiling as a feasible and effective option for preserving parent vessel integrity while securing the dissecting pseudoaneurysm.
